# Designing aesthetically authentic, restorative, and socially interactive environments for retirement homes: impacts on senior consumers’ relocation intention

**DOI:** 10.3389/fpsyg.2025.1541771

**Published:** 2025-10-08

**Authors:** Xianyao Ding, Xiaowen Xiong

**Affiliations:** ^1^School of Art and Design, Xihua University, Chengdu, China; ^2^School of Art, Sichuan Polytechnic University, Deyang, China

**Keywords:** aesthetic authenticity, restorative design, social interaction design, attitudinal beliefs, facilitating conditions, senior consumers’ relocation intentions

## Abstract

**Introduction:**

Drawing on the ecological model of aging and the technology acceptance model, this study investigates how three design factors—aesthetic authenticity, restorative design, and social interaction design—shape senior consumers’ relocation intentions in urban China.

**Methods:**

Survey data were collected from 331 seniors. Structural Equation Modeling (SEM) was applied to examine the direct effects, and PROCESS Macro Model 1 was employed to test the moderating effect.

**Results:**

Our analysis reveals a nuanced decision-making process. While all the above three factors positively influence attitudinal beliefs, their paths to shaping relocation intention differ. Aesthetic authenticity has a significant indirect effect through attitudinal beliefs, suggesting it is an intrinsic motivator. In contrast, the indirect effects of restorative and social interaction design are only significant when facilitating conditions (e.g., financial and social support) are high.

**Discussion:**

These findings contribute to gerontechnology and environmental design literature by demonstrating how foundational psychological needs and value-added features are weighed differently in seniors’ relocation decisions. We also provide practical implications for stakeholders on designing and marketing retirement communities within China’s evolving socio-cultural landscape.

## 1 Introduction

As a global phenomenon, the aging population has brought significant social, economic, and business implications. According to the World Health Organization, China has experienced rapid demographic aging since 2000, with an estimated 402 million of over 60 years old population by 2040, accounting for 28% of the total population.^1^ Such a demographic change results from declining birth rates, improving healthcare, and longer life expectancies (Bacanoiu and Danoiu, 2022; Mangoni, 2014).

While the traditional living arrangement of multigenerational household have supported senior people for a long time in China (Xi et al., 2023), this model is now under significant strain. Societal shifts such as urbanization and family structure changes have increasingly challenged the variability of this arrangement (Wang, 2018). First, the one-child policy has led to smaller core families, with fewer adult children looking after senior people. Second, many of those adult children relocating to developed areas for career development (Zhang et al., 2022). As such, the traditional intergenerational support that seniors have relied on becomes weaker, leading to increased social isolation and negative impacts on wellbeing (Cornwell and Waite, 2009; Kane and Choi, 1999).

Those challenges have driven government agencies to provide policies and initiatives that stimulated private developers to develop retirement communities providing different levels of services, such as healthcare services, recreational activities, dining facilities, and sometimes even on-site medical clinics or hospitals (Hu et al., 2021; Zhang and Yang, 2019). Retirement communities can cater to the unique needs and preferences of older Chinese consumers with comprehensive care services (Meng et al., 2020). According to China’s Ministry of Civil Affairs, the country had a total of 204,000 elderly care institutions in 2019 (Shan et al., 2021).

Previous research has identified several predictors in influencing senior consumers’ relocation intention to retirement homes, including availability and quality of services and facilities, government promotions (Cheng et al., 2011; Qin, 2020), perceptions of home, senior people’s health conditions, and neighborhood sociability (Crisp et al., 2013). Indeed, retirement homes should satisfy not only the functions but also personal meaningfulness for a senior consumer (Eshelman and Evans, 2002). However, researchers have identified design issues that impede the satisfaction of senior consumer expectations of a homely and comfortable environment. First, the aesthetic design resembles hospitals or nursing homes rather than inviting homes (Cutler, 2007). While practicality is important, the negligence of ambiance in retirement home design can lead to feelings of depersonalization and discomfort (Regnier, 2018).

Second, while medical resources are essential for clinical care, senior consumers may need more restorative resources that encompass a broader scope of services tailored to enhance their physical, emotional, and social lives (Cohen-Mansfield et al., 2012). While practitioners have highlighted the importance of long-term care facilities and services such as psychiatric treatment, addiction and eating disorder treatment, not much academic research have covered the role of these services in shaping senior consumers’ relocation intentions. This is despite the fact that those resources are essential for senior consumers to maintain overall health and quality of life (Ayalon, 2016).

Third, senior consumers in retirement communities have a need to interact not only with other seniors but also with people in society (Ten Bruggencate et al., 2018). This might be affected by the design of retirement homes with limited mobility and opportunities for social interaction. Scholars (Ying et al., 2021) have stressed the essential role of social connectedness in designing age-friendly environment, yet not much consumer behavior studies have examined its impact on senior consumers’ relocation intentions.

In response to these challenges, researchers have promoted gerontechnology, i.e., adopting technologies to enhance the living experiences of older adults (Gao et al., 2023; Piau et al., 2014). However, existing research in gerontechnology often focuses on technologies (Grigorovich et al., 2022; Merkel and Kucharski, 2019), rather than their integration into the design of retirement homes. While technologies have shown potential in improving various aspects of senior consumers’ lives (Huang and Oteng, 2023), their full potential can only be realized when seamlessly integrated into the overall design of retirement homes.

As a result, this study takes a design perspective to examine how gerontechnology is integrated into retirement home design to address the above-mentioned issues and create living environments that are not only aesthetically pleasing and socially engaging but also restorative to meet the needs of senior consumers. We adopt the ecological model of aging to examine how the design considerations of aesthetic authenticity, restorative health design, and social interaction design affect senior consumers’ relocation intention to retirement homes. This model integrates the individual, interpersonal, environmental and societal factors that influence senior people’s decision-making processes (Peters et al., 2022; Zheng and Yang, 2019), it particularly highlights the importance of interpersonal relationships and social support networks for wellbeing. To understand this complex decision-making process, we propose an integrated framework. We use the ecological model of aging as our foundational context, as it identifies the critical individual, environmental, and social factors that shape seniors’ experiences. Within this broad context, we incorporate core principles from the technology acceptance model (TAM) (Guner and Acarturk, 2020) to explain the specific cognitive pathway through which perceptions of a new environment lead to behavioral intentions. This allows us to examine not only what design factors matter, but how they shape the attitudinal beliefs that drive the intention to relocate.

Moreover, the ecological model of aging highlights interpersonal relationships and social support networks in older adults’ wellbeing (Shin and Park, 2022). Drawing on previous studies (Bratt and Fagerström, 2023; Lak et al., 2020), we further integrate attitudinal beliefs about the three design considerations as well as facilitating conditions into the framework. The objective of this study is to formulate a conceptual model to examine the senior consumers’ perceptions of the aforementioned design considerations. Specifically, it aims to (1) investigate how aesthetic authenticity, restorative health design, and social interaction design influence senior consumers’ attitudinal beliefs regarding retirement homes, and (2) explore the moderating role of facilitating conditions in shaping the relationship between attitudinal beliefs about retirement homes and the intention to utilize them.

The rest of this paper is structured as follows. Section 2 presents the theoretical background and hypotheses that form the conceptual model of this study, followed by the methods and results sections which introduced the data collection process and findings on hypothesis testing. Section 5 discusses the theoretical and managerial implications, as well as the limitations and future research suggestions, with Section 6 concluding this paper.

## 2 Theoretical background and hypothesis development

### 2.1 Senior consumers’ relocation intention to retirement homes

Senior consumers’ intention to accept retirement homes has caught increasing academic and practical interest. Previous studies have examined the predictors of senior consumers’ attitudes and behavior intentions, including location, amenities, and healthcare services (Ng et al., 2020; Sixsmith and Sixsmith, 2008). Although these factors address the practical aspects of senior homes, they have not fully captured the complex and nuanced needs of senior consumers, such as authentic replication of previous living surroundings, affording social interactions, and meeting restorative needs beyond medical facilities.

We consider how authenticity, social interaction, and restorative design are tailored to address senior consumers’ emotional, social, and psychological needs of aging, ultimately influencing their relocation intention to retirement homes. In doing so, we extend the functionality approach (Choi et al., 2020) by recognizing that the design of retirement homes should ensure aesthetically authentic living environments, with social interactions with not only senior neighbors but also the broader community, and holistic restorative support, thereby satisfying the diverse and evolving needs of senior consumers and contributing to their physical, emotional, and social wellbeing. The following sections discusses the theoretical lens to examine the association between key constructs.

### 2.2 Ecological model of aging

The ecological model of aging provides a valuable lens to examine the dynamic interplay between individuals and their environments as they age. Originated in the context of gerontology, this model posits that one’s health and wellbeing in senior age is shaped by physical, social, and cultural contexts, recognizing that aging occurs within a complex ecological system (Bronfenbrenner, 1994; Wahl et al., 2012).

This model also sheds light on design of retirement homes that by providing aesthetically authenticity, restorative services, and social interaction opportunities to ensure effective interconnectedness between senior consumers and their environments. Specifically, aesthetically authentic design resonates with the micro-level interactions between individuals and their immediate surroundings (Wahl and Oswald, 2010). Likewise, restorative design catering for seniors consumers’ physical and emotional wellbeing considers meso-level influences of social networks and community resources (Gitlin et al., 2016). Finally, social interactive design aimed to form meaningful social connections correspond to the macro-level impacts of cultural norms, values, and societal attitudes toward aging (Sixsmith and Sixsmith, 2008). Moreover, this design corresponds to the social connection opportunities that enable senior consumers to blend into the traditional values of retired life.

While the ecological model of aging can help identify the multi-level factors that influence wellbeing, it is less specific about the precise cognitive mechanisms that translate perceptions of an environment into a behavioral decision, such as the intention to relocate. To address this gap, we integrate core tenets of the TAM. TAM provides a parsimonious and highly validated framework for explaining how user perceptions of a system (in this case, the designed environment of a retirement home) forms an attitude, which in turn predicts the intention to use that system.

In our integrated theoretical framework, we posit: the ecological model of aging provides the overarching context, defining the key environmental design factors—aesthetic authenticity (micro-level), restorative design (meso-level), and social interaction design (macro-level)—as critical inputs shaping a senior’s experience. The cognitive processing of these factors is then explained through the logic of TAM. In our model, the perceptions of these design features collectively inform seniors’ attitudinal beliefs, a construct analogous to TAM’s attitude toward using. This attitude then drives relocation intention, which is analogous to TAM’s behavioral intention. Therefore, TAM provides the necessary explanatory power for the psychological process that links environmental perception to relocation intention, operating within the broader framework supplied by the ecological model.

### 2.3 Aesthetic authenticity and attitudinal beliefs

In this study, we define aesthetic authenticity as the design and establishment of retirement homes that align with senior consumers’ previous living experiences and enable them to feel a genuine connection to their new surroundings (Wise et al., 2019). Examples of such design include the incorporation of personal touches such as family photographs and customized living spaces (Tan et al., 2022; Van Hoof et al., 2015). Previous studies have confirmed that senior consumers tend to live in environments that form a sense of home, i.e., making them feel familiar and homely rather than characterized with institutional or clinical settings (Feldmann, 2020; Van Hoof et al., 2016).

According to Lawton (1980), living environments that meet senior consumers’ existing values, preferences, and life histories could generate a sense of continuity and coherence, as well as positive attitudes. Aesthetically authentic retirement homes can foster provide feelings of familiarity and connection and mitigate the feelings of displacement (Golant, 2008). Moreover, aesthetically pleasant living environments can positively affect senior consumers’ mood and cognitive functioning (Lui et al., 2009). For instance, incorporating tactics such as familiar furnishings, natural lighting, and homely décor, retirement home designers can create environments that elicit feelings of comfort, security, and belonging among senior consumers (Lui et al., 2009). Moreover, such environments may enhance senior consumers’ perceptions of the retirement home as a place where they can maintain their autonomy, dignity, and sense of identity (Lawton, 1980). As a result, aesthetic authenticity may lead senior consumers to form desirable attitudinal beliefs toward retirement homes, perceiving them as comfortable and contributing to their wellbeing. Therefore, we propose:

*Hypothesis 1:* Aesthetic authenticity is positively associated with senior consumers’ attitudinal beliefs toward retirement homes.

### 2.4 Restorative design and attitudinal beliefs

Drawing on existing studies (Fumagalli et al., 2020; Roe and Roe, 2018), we define restorative design in retirement homes as the tactical manipulation of architectural, interior, and environmental elements to enhance the physical, cognitive, and emotional wellbeing of senior consumers. Unlike medical facilities that focused on treatment, restorative design focuses on establishing living environments that can contribute to senior consumers’ recovery, rehabilitation, and overall wellness (Ulrich et al., 1991). For instance, technologies could provide experiences of walking in virtual environments to enhance nature connectedness and reduce stress (Chan et al., 2023). The sensory effects created by technologies can foster nature-like environments where senior consumers can have timely access to restorative resources, such as natural light and sounds, pleasant fragrances, soft textures (Karaman and Selcuk, 2021). In such environments, senior consumers can recover from attention fatigue and chronic stress; these could improve their daily functioning and mental health (Grave et al., 2023).

Moreover, studies have suggested how ergonomic furniture and therapeutic amenities can enhance senior consumers’ physical wellbeing by enabling them to live with safety and comfort (Gitlin et al., 2014). Moreover, natural elements such as plants, water features and lighting systems can make the spaces feel more calming, comfortable, and nurturing (Detweiler et al., 2012; Morales-Bravo and Navarrete-Hernandez, 2022; Rappe, 2005). In other words, retirement homes featured with a restorative design can provide opportunities for senior consumers to engage in activities that support their daily activities, mental health, and sense of autonomy, leading to a sense vitality within retirement homes. These elements can collectively lead senior consumers to feel more positive about the living environments in retirement homes and reduce their misgivings about the clinical and confining feelings of traditional nursing homes. As a result, the following hypotheses can be proposed:

*Hypothesis 2:* Restorative design is positively related to senior consumers’ attitudinal beliefs about retirement homes.

### 2.5 Social interaction design and attitudinal beliefs

Drawing on aging studies (Köttl et al., 2021; Prattley et al., 2020; Wahl and Oswald, 2010), this study defines social interaction design as the deliberate arrangement of physical spaces, facilities, and activities to provide opportunities social engagement between senior consumers, their neighbors, and other social members. Examples of social interaction design include communal areas and amenities (Chen et al., 2020), social technologies such as social media platforms, video conferencing and gaming platforms (Liu and Tamura, 2020; Liu et al., 2020) and the development of diverse activities tailored to meet the social needs and preferences of senior consumers in these facilities (Tyvimaa, 2011).

Well-designed social interaction facilities allow senior consumers to enjoy the social engagement which is related to positive attitudes toward their living environment (Wahl and Oswald, 2010). Social interaction design highlights the need to meet senior consumers’ social and emotional needs by designing environments that support social engagement, thus leading to improved attitudes among senior consumers (Chaudhury et al., 2016). The communal spaces and technologies that facilitate physical and virtual activities, senior consumers may develop a sense of belonging and connection to not only their neighbors but also other individuals in society (Glover, 2017).

Research indicates that social interaction is an integral to cognitive functioning, emotional health, and overall life satisfaction of senior consumers (Seeman et al., 2001). Retirement homes with a social interaction design allows senior consumers to form meaningful connections, participate in group activities, and engage in shared hobbies (Sixsmith and Sixsmith, 2008). Such environments can mitigate the feelings of isolation and loneliness documented in senior consumer studies (Chu et al., 2020; Pettigrew, 2007). Environments with supportive social interactions can enhance seniors consumers’ emotional wellbeing and affect senior consumers’ attitudinal beliefs about retirement homes (Park et al., 2013), perceiving those homes to support their social, emotional, and psychological needs. Therefore, we propose the following hypothesis:

*Hypothesis 3:* Social interaction design is positively related to senior consumers’ attitudinal beliefs about retirement homes.

### 2.6 Attitudinal beliefs and relocation intention

Attitudinal beliefs include the cognitive, emotional, and behavioral evaluations that individuals develop toward specific products or services (Authority, 2020; Gibler et al., 1998). In the context of retirement homes, these beliefs include cognitive and emotional assessments developed from senior consumers’ perceptions of autonomy, comfort, social opportunities, and overall life quality (Sixsmith and Sixsmith, 2008). Senior consumers with positive attitudes may believe that retirement homes can provide the amenities, services, social activities, and personalized environments that can ensure their wellbeing in the senior age (Phillipson and Smith, 2005). Moreover, positive attitudes toward retirement homes suggest senior consumers’ positive perception that they are able to maintain an independent and autonomous lifestyle that aligns with their self-reliance value (Gitlin et al., 2016). Previous studies have associated positive perceptions and attitudes about the various amenities, services, environments, and other benefits available in retirement homes with senior consumers’ intention to relocate (Krout et al., 2002; Lowies et al., 2020). We further propose the following hypothesis:

*Hypothesis 4:* There is a positive association between attitudinal beliefs and relocation intention among senior consumers.

### 2.7 Facilitating conditions

Despite its important role in shaping consumer behaviors, positive attitudinal beliefs alone may not be sufficient to stimulate relocation intention, unless supported by facilitating conditions. Indeed, senior consumers who are positive about retirement homes may still face significant barriers, such as limited financial resources to cover the relevant costs (Gitlin et al., 2016). Likewise, lack of support from family members or friends, and the traditional social norms (filial piety) may reduce senior consumers’ confidence in making life transitions in retirement homes, making them hesitant and isolated about relocation.

Facilitating conditions refer to the existence of factor or circumstances that supports the adoption or use of a particular service or product (Venkatesh et al., 2003). In the context of this study, facilitating conditions involve the factors that support senior consumers to develop intentions or make decisions to relocate to retirement homes. The literature has documented a number of such factors, such as financial affordability, social support from families and friends, and cultural norms about retirement homes (Eriksson et al., 2022; Franco et al., 2021).

Facilitating conditions are important for consumers to make decisions shaping individuals’ decision-making processes and behaviors (Abbad, 2021). Indeed, senior consumers’ intention to relocate to a retirement home depends on the interplay of financial, social, and cultural factors that can be either facilitating or discouraging (Chaulagain et al., 2021). For instance, senior consumers with positive attitudinal beliefs toward retirement homes may be more inclined to relocate if they perceive readily available transportation services that enable them to blend into local communities rather than being isolated at home. In other words, facilitating conditions can moderate the degree to which senior consumers’ attitudinal beliefs translate into relocation intentions. As a result, the following hypothesis can be predicted:

*Hypothesis 5:* Facilitating conditions moderate the association between senior consumers’ attitudinal beliefs and relocation intention to retirement homes.

## 3 Methods

### 3.1 Sampling

This study examined the hypothesized relationships by surveying senior consumers’ relocating intention from Beijing, Shanghai, Hangzhou, and Chengdu in China (The high-quality development of retirement homes determined the chosen cities). The survey employed the online survey platform ‘WENJUANXING’ (a popular website for questionnaire surveys in China). This study collected 350 responses; after excluding 19 invalid questionnaires, the sample for analysis consisted of 331 responses (94.57% of the total). 48.0% of the respondents are male (*n* = 159), 52.0% of the respondents are female (*n* = 172). 76 years old and above (44.7%) has the highest per cent among the three groups, followed by 66–75 years old (34.1%), and 55–65 years old (21.1%). Table 1 presents the results of those respondents.

**Table 1:** Demographic description.

Variable		Frequency	Percentage
Gender	Male	159	48.0%
	Female	172	52.0%
Age	55–65 years old	70	21.1%
	66–75 years old	113	34.1%
	76 years old and above	148	44.7%
City	Beijing	83	25.1%
	Shanghai	106	32.0%
	Hangzhou	80	24.2%
	Chengdu	62	18.7%

### 3.2 Measures

This study included six constructs: aesthetic authenticity, restorative design (with four secondary factors: fascination, being away, coherence, and scope), social interaction design, attitudinal beliefs, facilitating conditions, and relocation intention. All measurement items were sourced from existing literature and first adapted to fit the specific context of senior consumers’ perceptions of retirement home environments.

Following this adaptation, the scale items were reviewed by an expert panel on content validity, clarity, and contextual relevance. The panel consisted of four senior retirement home managers and three academic professors with expertise in gerontology and environmental psychology. Feedback from the panel was used to refine the wording of several items. Only after adaptation and expert review were the final items translated into Chinese by one author and back-translated into English by another to ensure semantic equivalence. This allowed us to maintain semantic equivalence throughout the translation process.

This allowed us to maintain semantic equivalence throughout the translation process. A 5-point Likert scale was used for each measurement item. Three items were adapted from Tran and Keng (2018) to measure aesthetic authenticity (AA). The sample items included “The design and decor of the retirement home feels familiar, comfortable, and reminiscent of my previous living environments.” Eleven items were adapted to assess the restorative design, which was comprised of four secondary factors: fascination (three items), being removed (three items), coherence (three items), and scope (two items), as defined by Pasini et al. (2014). The sample items included “I find the design of this retirement home to be captivating,” “I feel distanced from disturbances when I am in this retirement home,” and “I feel a sense of order and harmony in the design of this retirement home.” Social interaction design was measured by adapting six items taken from Anderberg et al. (2021). Sample items included “I feel a sense of belonging and inclusion in the social fabric of this retirement home and neighborhood,” “The design of this retirement home provides me with opportunities to engage in activities that are meaningful to me,” and “I find it easy to stay connected with friends and acquaintances from this retirement home.” Attitudinal beliefs was measured by adapting three items taken from Chen and Lou (2020). Sample items included “I think the design of the retirement home is well-planned and enhances the quality of life for its residents.” Facilitating conditions (three items) and relocation intention (three items) were adapted from Zin et al. (2023). Sample items included “I have sufficient financial resources to live in a retired home,” and “I would relocate to a retired home if given the opportunity.”

### 3.3 Data analysis

Initially, a measurement model was developed utilizing AMOS 24.0 to assess confirmatory factor analysis (CFA). Subsequently, SPSS 25.0 was employed to conduct reliability and correlation analyses. To reduce the potential influence of common method bias, we employed Harman’s single-factor test. We implemented a structural model to examine the direct effects of using AMOS. For analyzing the moderating effect, we utilized PROCESS Macro Model 1. The simple slope of the moderating effect was plotted according to the suggestion by Aiken and West (1991).

## 4 Results

### 4.1 Common method bias

**Figure 1:**
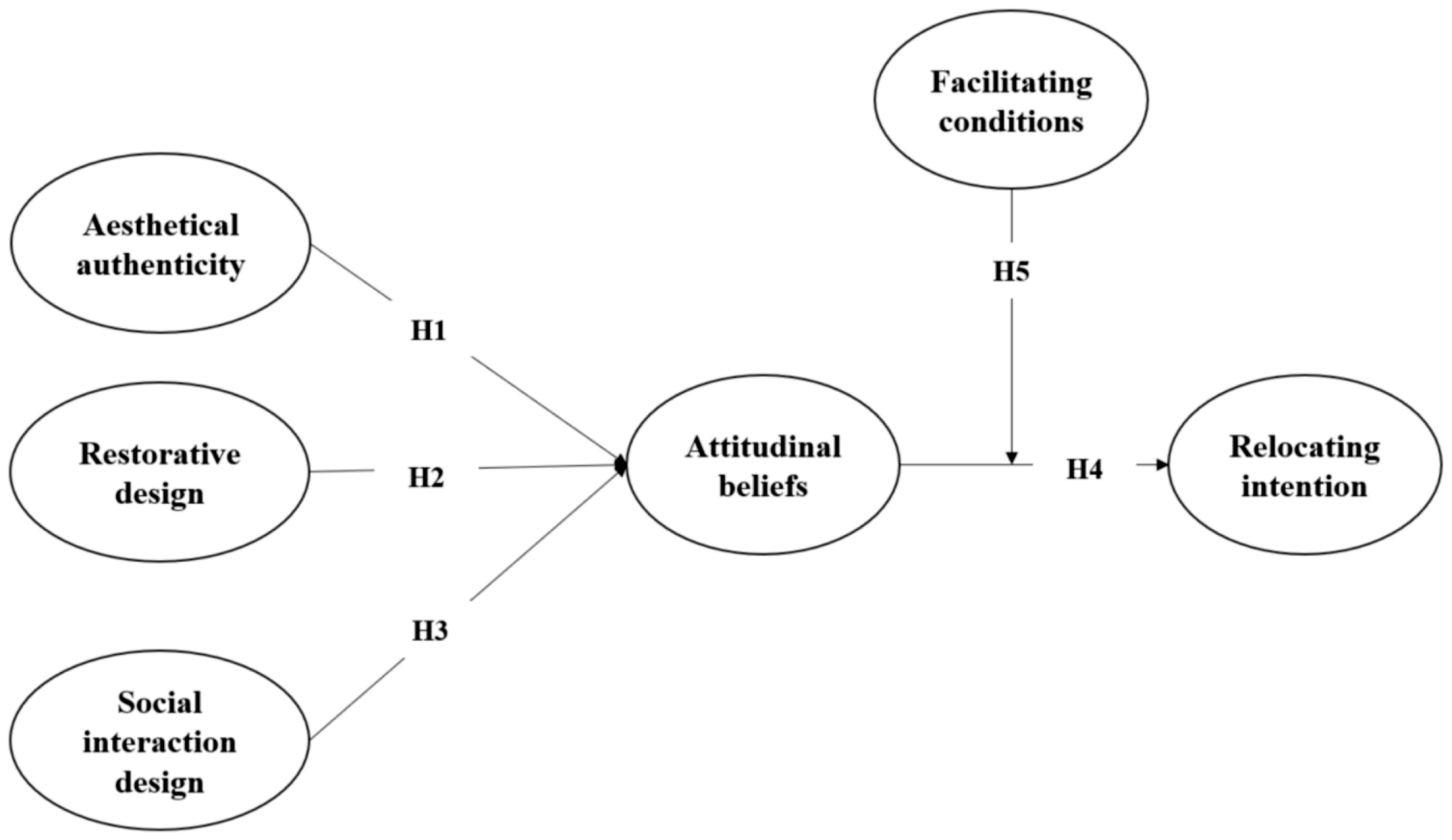
Conceptual model.

Harman’s single factor test was used to examine the issue of common method bias. In the result of exploratory factor analysis, the first factor that has not been rotated explains less than 40% of the variance (33.21% of 73.65%). Therefore, our findings had no serious common method bias (Figure 1).

### 4.2 Reliability and convergent validity

We first conducted a confirmatory factor analysis in AMOS. The reliability and validity were first examined to test the measurement model. As shown in Table 2, the factor loadings of the items are all higher than the required 0.70. Cronbach’s alpha and composite reliability (CR) were used to evaluate internal consistency reliability. The Cronbach’s Alpha and the CR of each variable are larger than the required 0.70 level, confirming the internal consistency and reliability. The model fit of the measurement model showed that *χ*^2^/df (chi-square divided by the value of the degree of freedom) = 1.072, CFI (comparative fit index) = 0.995, TLI (Tucker–Lewis index) = 0.995, RMR (root-mean-square residual) = 0.051, RMSEA (root mean square error of approximation) = 0.015, indicating a good fitness of the collected data with the measurement model. Furthermore, we also conducted an additional CFA comparing a single-factor model of ‘Restorative Design’ against a higher-order model with its four theoretical sub-dimensions. The results showed a significantly better fit for the higher-order model, justifying our conceptualization (One-factor model: *χ*^2^/df = 11.904, CFI = 0.695, TLI = 0.619, RMR = 0.151, RMSEA = 0.182; Higher-order model: *χ*^2^/df = 1.208, CFI = 0.995, TLI = 0.993, RMR = 0.048, RMSEA = 0.025). Average variance extracted (AVE) was utilized to evaluate convergent and discriminant validity. As shown in Table 2, all of the AVE values are greater than the required 0.5 level, confirming the convergent validity (Fornell and Larcker, 1981). According to Table 3, the correlations between the two constructs are all less than the square root of the construct’s AVE, which satisfies the criterion and confirms the discriminant validity.

**Table 2:** Result of reliability and convergent validity.

Variable	Item	Factor loading	CR	AVE	Cronbach’s Alpha
Restorative design	Fascination	0.824	0.809	0.518	0.864
	Being Away	0.735			
	Coherence	0.709			
	Scope	0.591			
Aesthetic authenticity	AA1	0.768	0.829	0.617	0.828
	AA2	0.798			
	AA3	0.790			
Social interaction design	SI1	0.788	0.929	0.687	0.929
	SI2	0.900			
	SI3	0.848			
	SI4	0.806			
	SI5	0.825			
	SI6	0.800			
Attitudinal beliefs	AB1	0.832	0.892	0.734	0.889
	AB2	0.805			
	AB3	0.929			
Relocation intention	RI1	0.848	0.896	0.743	0.894
	RI2	0.915			
	RI3	0.820			
Attitudinal beliefs	FC1	0.770	0.833	0.624	0.833
	FC2	0.797			
	FC3	0.803			

**Table 3:** Correlation and discriminant validity.

Constructs	Mean	SD	1	2	3	4	5	6	7	8	9
1. Gender	1.52	0.50	—								
2. Age	2.24	0.78	0.082	—							
3. City	2.37	1.05	−0.103	0.005	—						
4. Aesthetical authenticity	3.82	1.00	0.083	−0.096	−0.015	0.785					
5. Restorative design	3.69	0.78	0.008	0.004	0.038	0.464^**^	0.720				
6. Social interaction design	3.46	1.05	−0.067	−0.062	0.055	0.449^**^	0.532^**^	0.829			
7. Attitudinal beliefs	3.71	1.02	0.018	−0.043	−0.001	0.429^**^	0.449^**^	0.459^**^	0.857		
8. Facilitating conditions	3.70	1.02	0.055	0.023	−0.017	0.195^**^	0.237^**^	0.246^**^	0.185^**^	0.790	
9. Relocation intention	3.74	1.00	0.021	0.024	−0.024	0.340^**^	0.342^**^	0.394^**^	0.250^**^	0.380^**^	0.862

** *p* < 0.01. The square root of AVE is on the diagonal.

### 4.3 Hypothesis testing

**Figure 2:**
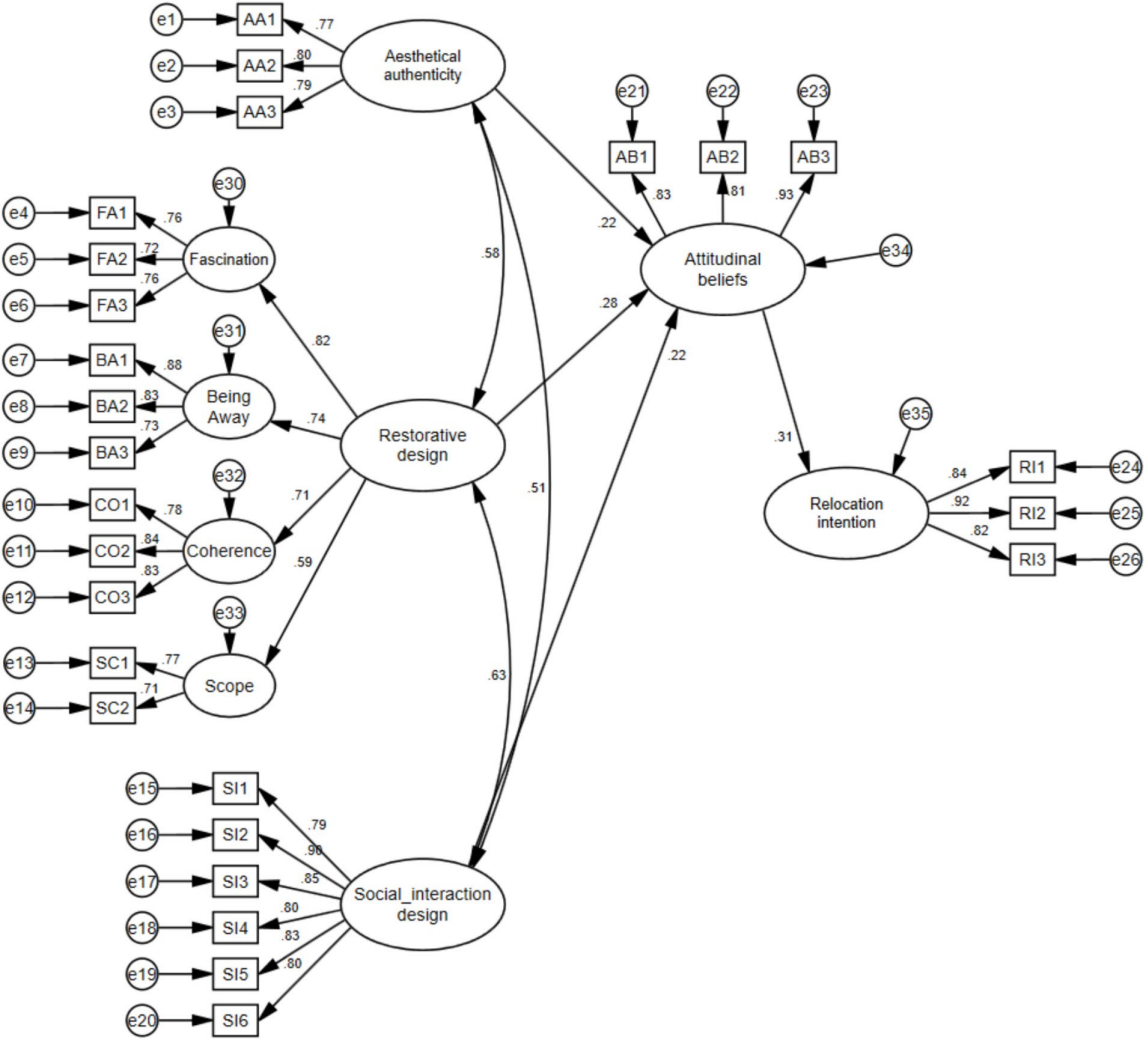
Structural model.

We used the structural model (see Figure 2) with AMOS 24.0 to test direct effects. The model fit of the structural model showed that *χ*^2^/df = 1.238, CFI = 0.986, TLI = 0.984, RMR = 0.097, RMSEA = 0.027, indicating a good fitness of the collected data with the structural model. The results of direct effects (Table 4) show that aesthetic authenticity (*β* = 0.219, *p* < 0.05), restorative design (*β* = 0.281, *p* < 0.05), and social interaction design (*β* = 0.218, *p* < 0.05) both have significant positive effects on attitudinal beliefs. Attitudinal beliefs (*β* = 0.314, *p* < 0.05) has a significant positive effect on relocation intention. Thus, H1–H4 are supported.

**Table 4:** Results of direct effects.

Path	STD. Estimate	S.E.	C.R.	*p*	Support or not
Attitudinal beliefs ← Aesthetic authenticity	0.219	0.081	2.954	0.003	Supported
Attitudinal beliefs ← Restorative design	0.281	0.117	3.085	0.002	Supported
Attitudinal beliefs ← Social interaction design	0.218	0.071	2.989	0.003	Supported
Relocation intention ← Attitudinal beliefs	0.314	0.055	5.225	^***^	Supported

*** *p* < 0.001.

**Figure 3:**
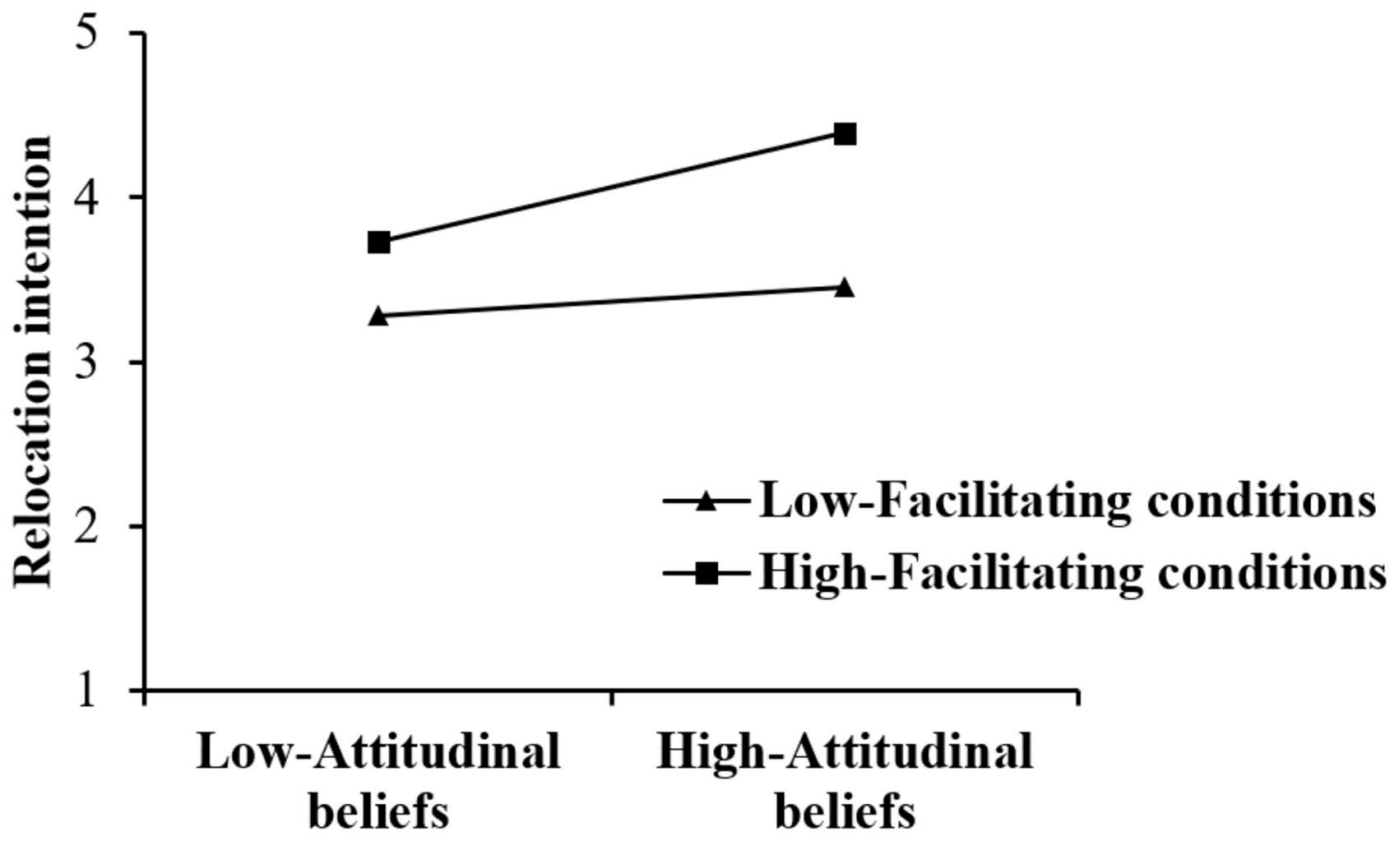
Result of moderating effect.

PROCESS Macro Model 1 was utilized for moderating effect testing. According to Table 5, the moderating effect of facilitating conditions on the relationship between attitudinal beliefs and relocation intention is significant, that is, when facilitating conditions is at a high level (Mean + 1SD), the relationship between attitudinal beliefs and relocation intention is stronger than it is at a low level (Mean − 1SD) (a simple slope plot presented in Figure 3). Thus, H5 is supported.

**Table 5:** Results of moderating effect.

Facilitating conditions	*β*	se	*t*	*p*	LLCI	ULCI
Mean − 1SD	0.086	0.062	1.394	0.164	−0.035	0.208
Mean + 1SD	0.323	0.074	4.359	0.000	0.177	0.469

Additionally, we conducted mediation (model 4) and moderated mediation analysis (model 14) to confirm the indirect effects of attitudinal beliefs. As illustrated in Table 6, attitudinal beliefs has a significant mediating effect on the relationship between aesthetic authenticity and relocation intention (effect = 0.055, LLCI = 0.002, ULCI = 0.110); the mediating effects of attitudinal beliefs on the relationship between restorative design (effect = 0.069, LLCI = 0.000, ULCI = 0.143), social interaction design (effect = 0.038, LLCI = −0.018, ULCI = 0.096) and relocation intention are not significant. Contrary to the results of mediation results, attitudinal beliefs has significant moderated mediating effects among restorative design (effect = 0.047, LLCI = 0.041, ULCI = 0.226) and relocation intention; social interaction design (effect = 0.037, LLCI = 0.016, ULCI = 0.159) and relocation intention.

**Table 6:** Mediation and moderated mediation results.

Path	Facilitating conditions	Effect	BootSE	BootLLCI	BootULCI
Aesthetic authenticity-Attitudinal beliefs-Relocation intention	–	0.055	0.028	0.002	0.110
Restorative design-Attitudinal beliefs-Relocation intention	–	0.069	0.036	0.000	0.143
Social interaction design-Attitudinal beliefs-Relocation intention	–	0.038	0.029	−0.018	0.096
Aesthetic authenticity-Attitudinal beliefs-Relocation intention	Mean − 1SD	0.015	0.033	−0.046	0.084
	Mean + 1SD	0.094	0.034	0.023	0.157
	Index = 0.047		0.025	−0.005	0.093
Restorative design-Attitudinal beliefs-Relocation intention	Mean − 1SD	0.016	0.042	−0.062	0.105
	Mean + 1SD	0.133	0.047	0.041	0.226
	Index = 0.07		0.034	0.001	0.132
Social interaction design-Attitudinal beliefs-Relocation intention	Mean − 1SD	−0.003	0.033	−0.064	0.066
	Mean + 1SD	0.090	0.037	0.016	0.159
	Index = 0.056		0.025	0.002	0.102

## 5 Discussion

This study aims to investigate how aesthetic authenticity, restorative health design, and social interaction design influence senior consumers’ attitudinal beliefs about retirement homes. It also seeks to unravel how facilitating conditions moderate the association between these beliefs and senior consumers’ intentions to relocate to retirement homes. In doing so, we incorporated the designing factors in shaping senior consumers’ decision-making processes, thus unraveling their perceptions, attitudes, and intentions about retirement home relocation.

We found positive association between aesthetic authenticity and senior consumers’ attitudinal beliefs. This concurs with the literature (Wahl and Oswald, 2010) regarding the importance of environmental factors in consumer perceptions. However, our study probed deeper into the relationship between aesthetic authenticity and attitudinal beliefs, highlighting how the design aspects of retirement homes influence senior consumers’ perceptions of authenticity. In doing so, we extend the functionality approach (Choi et al., 2020) by highlighting the role of aesthetic authenticity in retirement home design.

We found positive correlations between restorative design and senior consumer’ attitudinal beliefs about retirement homes. This concurs with studies (Grave et al., 2023) highlighting the essential role of restorative resources in shaping senior consumers’ wellbeing. However, unlike the assumption that retirement homes have to be located near nature, we explored the possibility of integrating restorative resources into the design of retirement homes. We also found social interaction design positively associated with attitudinal beliefs; this aligns with Sixsmith and Sixsmith (2008) on the importance of social engagement in later life. We extend studies on social interaction and gerontechnology (Grigorovich et al., 2022; Ying et al., 2021) through a design perspective. Our results suggest how embedding social technologies into the built environment can facilitate opportunities for social engagement among senior homes.

Our mediation analysis suggests that ‘attitudinal beliefs’ acts as a mediator only for the path from aesthetic authenticity to relocation intention. This suggests that creating a familiar, home-like environment directly fosters positive attitudes, which in turn drive relocation intentions. Interestingly, this direct mediation was not significant for restorative and social interaction design. Instead, the moderated mediation results show that their indirect effects only become significant when facilitating conditions are high. For example, the positive influence of restorative design on relocation intention through attitudinal beliefs is only activated when seniors perceive they have the financial and social support to move. While an authentic feeling is a powerful intrinsic motivator, the benefits of advanced restorative and social features are contingent upon practical enablers. These features may only translate into a relocation intention if the move is perceived as feasible.

Our results on the positive association between attitudinal beliefs and relocation intentions align with previous research on the role of attitudes on housing decisions among older adults (Phillipson and Smith, 2005). We examined the moderating role of facilitating conditions. Unlike previous studies on facilitating conditions in technology acceptance context (Venkatesh et al., 2003), this study examined the specific conditions that help interpret how the specific conditions interact with senior consumers’ attitudinal beliefs toward retirement homes, highlighting the complex interplay between cognitive, environmental, and contextual factors in shaping housing decisions among older adults.

### 5.1 Theoretical contributions

This study has several theoretical contributions. First, we applied the ecological model of aging in the context of retirement home relocation intentions. While this model involves the interaction between individuals and their broader environments (Bronfenbrenner, 1994), we applied it to the decision-cognitive processes of senior consumers considering relocation to retirement homes. By considering the influence of three design-specific predictors of attitudinal beliefs, and facilitating conditions within retirement home environments, we tailored the ecological perspective to interpret the complex interplay between individual perceptions and environmental factors in shaping senior consumers’ relocation intentions to retirement homes.

Second, we refine the application of TAM model context of senior housing by demonstrating that not all environmental factors operate through the same cognitive pathway (Guner and Acarturk, 2020) to encompass the built environment of retirement homes. Our finding that aesthetic authenticity has a direct and indirect effect on relocation intention through attitudes suggests that this factor acts as a core motivator. In contrast, the indirect effects of restorative and social interaction design were contingent upon facilitating conditions. This suggests that these factors are processed differently; they are valued, but their power to drive intention is only unlocked when practical barriers are removed. This contributes a more nuanced understanding of how foundational psychological needs (a sense of ‘home’) and value-added features are weighed in the decision-making process.

Third, we contribute to the gerontechnology literature (Gao et al., 2023) by highlighting the role of design features within retirement homes in shaping senior consumers’ perception of wellbeing and quality of life. In particular, we examine how technologies are integrated into retirement home design to develop solutions that enhance older adults’ autonomy, health, and social participation. Our findings on the positive role of aesthetical authenticity, restorative design, and social interaction design in shaping senior consumers’ attitudinal beliefs about retirement homes exemplified the relevance of design perspective in creating age-friendly retirement homes. These findings also confirmed the potential for gerontechnology applications to optimize retirement home design.

### 5.2 Practical implications

Our results present several implications for the relevant stakeholders in the planning, designing and management of retirement homes. First, our findings shed valuable insights for policymakers in retirement home initiatives. The importance role of aesthetic authenticity, restorative design, social interaction design, and facilitating conditions suggest that policymakers in China should develop guidelines that are sensitive to the nation’s unique demographic shifts, especially the weakening of traditional multigenerational households due to urbanization and past policies. Public advertising should present retirement homes not as a departure from filial piety norms, but as a practical solution that ensures professional care and social engagement for seniors in today’s society. This can help change stereotypes and align the concept of retirement communities with evolving Chinese family values. Moreover, policymakers can explore incentives such as to improve access to supportive services and resources, addressing barriers that may hinder seniors consumers’ relocation intentions (Gitlin et al., 2016).

Second, our findings offer a strategic roadmap for retirement home investors and designers. Our findings show that aesthetic authenticity should be considered a foundational design consideration. Creating a familiar, comfortable, and home-like environment is directly associated with positive attitudes and should be the first priority. In contrast, features related to restorative and social interaction design may only translate into relocation intention for seniors who have high levels of facilitating conditions (e.g., financial resources and family support). Therefore, marketing efforts promoting these advanced features should be coupled with practical support and information that addresses feasibility. For instance, marketing materials should include clear information on financing options, transportation services, and family integration programs to overcome the barriers of relocation.

Third, retirement home managers could collaborate with local communities to enhance social integration and resource access. For instance, recreational activities, education programs, and volunteer opportunities can promote a sense of belonging. Moreover, senior consumers can be invited to provide timely feedback to retirement home design. This could facilitate collaboration and co-creation between senior consumers, designers, and managers, and enhance the overall living quality in retirement homes.

### 5.3 Limitations and future research

Despite its contributions, this study is subject to limitations that require future research efforts. First, we used self-reported data, which is susceptible to potential biases. Responses about attitudinal beliefs and relocation intentions could be influenced by social desirability bias. Participants might provide answers they deem more socially acceptable rather than reflecting true feelings. Furthermore, since aesthetic authenticity involves comparing a new environment to previous living experiences, recall bias could affect how participants remember and report these feelings. Future studies could adopt longitudinal designs to track how intentions evolve and translate into actual relocation decisions over time. Moreover, mixed-methods that include in-depth qualitative interviews could help future research to triangulate data and validate the survey findings.

Second, our findings’ generalizability is limited by the study’s specific geographic and demographic scope, as well as our sampling method. We used an online survey platform, which may introduce a potential sampling bias due to the digital divide. This method may underrepresent seniors with limited digital literacy or access to technology, a concern that is particularly relevant for the oldest cohort of our sample (76 years and above). Our sample may hence skew toward individuals who are more technologically proficient and potentially more affluent than the senior population.

Furthermore, the sample was drawn from four economically developed Chinese cities (Beijing, Shanghai, Hangzhou, and Chengdu) noted for their high-quality retirement homes. The preferences and intentions of seniors in these urban, digitally-connected contexts may differ from those in less-developed Chinese cities. Additionally, senior consumers in other countries with different cultural norms (e.g., regarding filial piety), healthcare systems, and social support structures may form different preferences and intentions. Similarly, we did not categorize participants by health status or socioeconomic level, which can shape housing needs and preferences. Future research should compare cross-cultural constructs to test the model’s validity in different national contexts. Moreover, future studies could segment the senior population by health (e.g., independent versus assisted living needs) and financial situation to capture the diverse range of experiences within this demographic.

Third, we primarily examined individual-level factors (micro-level) and their social context (meso-level). We did not incorporate macro-level contextual influences that can shape relocation intentions. These include factors such as neighborhood characteristics (e.g., access to public transportation), local housing market dynamics, and the presence of supportive government policies. Future studies should employ a multi-level modeling approach to simultaneously assess the influence of individual perceptions and objective, neighborhood-level variables on seniors’ housing decisions.

## 6 Conclusion

Drawing on the ecological model of aging, we investigated the factors influencing senior consumers’ relocation intentions toward retirement homes in urban China. We find that aesthetic authenticity, restorative design, and social interaction design are positively associated with attitudinal beliefs, which predict relocation intentions. These findings are particularly significant in the socio-cultural context of China, the erosion of traditional family support systems coincides with a growing need for senior living solutions. We stress that for Chinese senior consumers, a retirement home should feel more than a care facility. Instead, it must be an authentic, restorative, and socially vibrant environment that meets their holistic needs.

## Data Availability

The data analyzed in this study is subject to the following licenses/restrictions: the raw data supporting the conclusions of this article will be made available by the authors, without undue reservation. Requests to access these datasets should be directed to xianyao.ding@mail.xhu.edu.cn.

## Footnotes

^1^ https://www.who.int/china/health-topics/ageing
